# Is repair of the protruded meninges sufficient for treatment of meningocele?

**DOI:** 10.1007/s00381-015-2874-4

**Published:** 2015-08-23

**Authors:** Song Yun-Hai, Bao Nan, Gao Ping-Ping, Yang Bo, Chen Cheng

**Affiliations:** Neurosurgery Department, Shanghai Children’s Medical Center Affiliated to the Medical School of Shanghai Jiaotong University, Shanghai, 200127 China

**Keywords:** Meningocele, Tethered cord syndrome, Diagnosis, Surgery

## Abstract

**Purpose:**

The present study aimed to investigate the relationship between meningocele and tethered cord syndrome, diagnosis of meningocele associated with tethered cord syndrome, and when to perform surgery and the best surgical procedure.

**Methods:**

Sixty-nine children with meningocele who were admitted to Shanghai Children’s Medical Center were analyzed. The relationship between meningocele and other lesions causing tethered cord syndrome was studied by combining magnetic resonance imaging (MRI) and intraoperative findings.

**Results:**

The MRI results and intraoperative findings showed that 67 children (97 %) had associated lesions such as tight filum terminale, fibrous band tethering, spinal cord or cauda equina adhesion, diastematomyelia, arachnoid cyst, and epidermoid cyst. The protruded meninges were repaired, and the intraspinal lesions were treated at the same time. Also, the tethered spinal cord was released. No neurological injuries were observed after surgery.

**Conclusions:**

The rate of meningocele associated with tethered cord syndrome is very high. MRI is necessary for the diagnosis of meningocele. Active surgical treatment is recommended immediately after definite diagnosis. During surgery, the surgeon should not only repair the protruded meninges but also explore the spinal canal and release the tethered cord.

## Introduction

Meningocele is a common congenital neural tube defect. It consists of a protrusion of the meninges through a bone defect in the spine towards dorsal, presacral, and paraspinal spaces, while the spinal cord is still inside the spinal canal [1]. In clinical practice, it is believed that the prognosis of patients with meningocele is excellent and simple surgical repair of the meninges is sufficient. However, it has been reported recently that meningocele is often associated with tethered cord syndrome and that symptoms of tethered cord syndrome gradually developed in a considerable portion of children who underwent meningocele repair [2, 3]. Since 2008, we have studied 69 children with meningocele and found that the rate of meningocele associated with tethered cord syndrome was very high. Herein, we provide an introduction on meningocele-associated diseases and their diagnosis, timing for surgery, and surgical approaches.

## Methods

### Clinical data

From January 2008 to December 2013, 69 children with meningocele were admitted to Shanghai Children’s Medical Center. Thirty-seven were males and 32 were females. They ranged in age from 3 days to 8 years (average, 9.5 months). Magnetic resonance imaging (MRI) was performed before surgery for all children to obtain a definite diagnosis. The surgical procedure included repair of the protruded meninges, removal of the spinal lesions, and release of the tethered spinal cord. Sixty-seven of the 69 children were followed up after surgery; two children were lost to follow-up. The mean follow-up period was 45.2 months (range, 8 months to 6 years). During follow-up, defecation and urination states, lower-extremity muscle power, and presence of foot deformities were observed.

## Results

### Clinical manifestations

The meningocele was located in the lumbosacral region in 56 children, in the thoracic region in 8, and in the cervical region in 5 children. Before surgery, 56 children had no symptoms but 13 had symptoms due to neurological damage. Sphincter function was impaired in 10 children, abnormally increased stool frequency with fecal leakage was observed in 6, and abnormal defecation together with acraturesis was noted in 4 children. Clubfoot deformity or talipes cavus associated with hypoesthesia and decreased muscle strength was observed in 3 children. Two of the 13 children with neurological symptoms had a history of simple meningocele repair. Both of these children had fecal and urinary incontinence or foot deformities 1 year before admission.

### Imaging examination

**Fig. 1:**
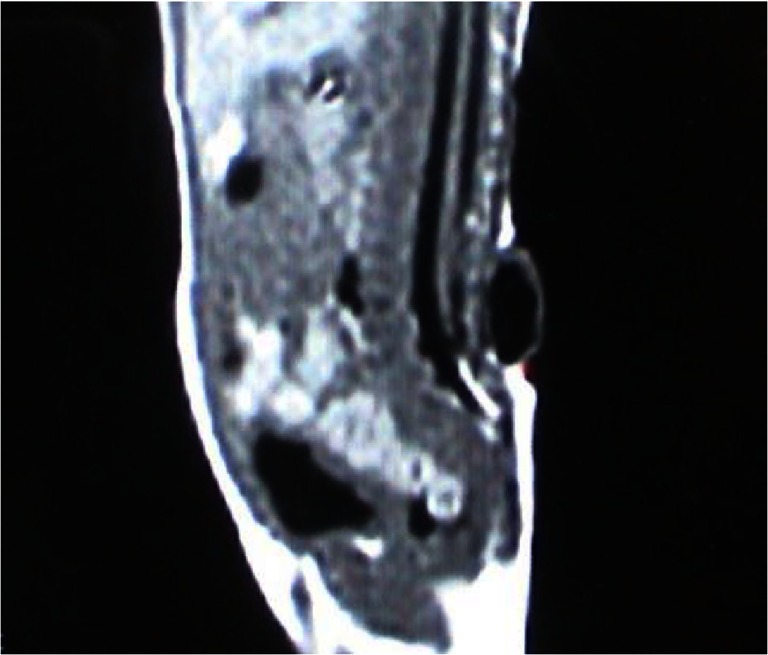
A lumbosacral meningocele: the conus medullaris is located at the level of L5, and fat invasion is seen in the filum terminale

**Fig. 2:**
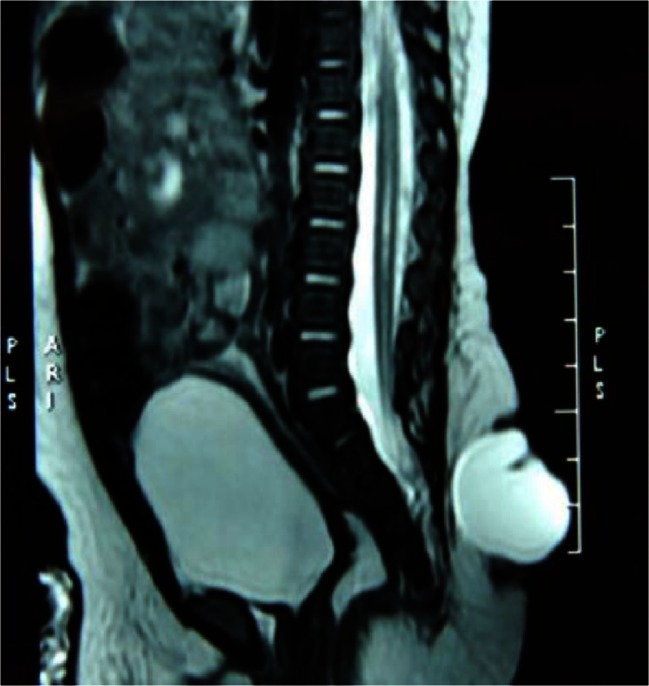
A sacral meningocele: the conus medullaris is located low at the level of L5, and there is associated syringomyelia

**Fig. 3:**
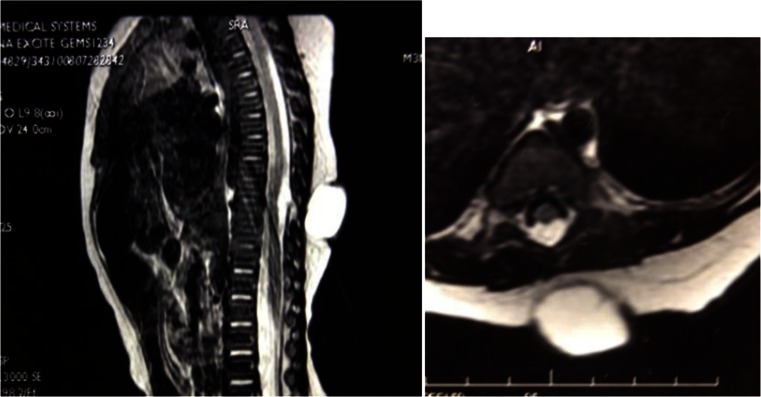
A thoracic meningocele: there is an arachnoid cyst located at the dorsal side of the spinal cord

The MRI results proved that all 69 children had a meningocele and that there was no neurological tissue within the protruded meningocele sac. However, a low-lying conus medullaris was observed in 43 children. Among these 43 children, 27 had associated lesions such as fat invasion in the terminal filament (Fig. 1); spinal cord lipoma, diastematomyelia, or syringomyelia (Fig. 2); or an arachnoid cyst located dorsal to the spinal cord (Fig. 3). The conus medullaris was in the normal location in the other 26 children. However, among these 26 children, there were 5 who had Chari malformation, syringomyelia, an arachnoid cyst, or an arachnoid cyst with an epidermoid cyst.

### Surgical findings

During surgery, the meningocele sac was dissected and cut open, and the spinal canal was expanded cephalically and caudally. Half of the spinous process was removed, and the meningocele sac was cut open from the base superiorly and inferiorly to expose the normal dural sac. The spinal canal was explored to search for the lesions shown on MRI. There were some other intradural lesions found which were not shown by MRI.

**Fig. 4:**
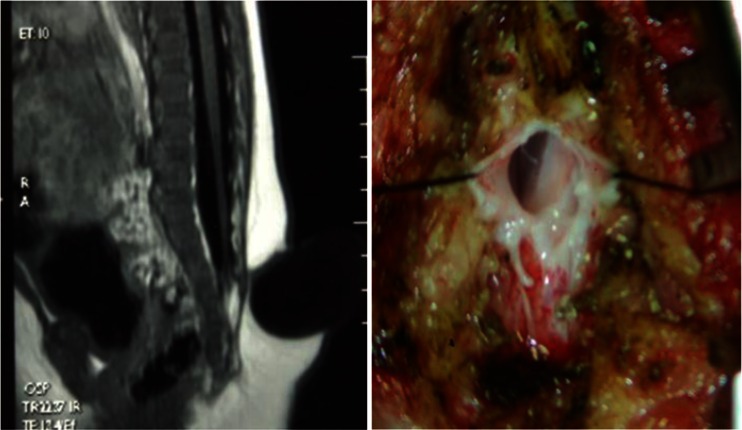
A sacral meningocele: the spinal cord is adhered to the dura mater at the base of the dural sac

**Fig. 5:**
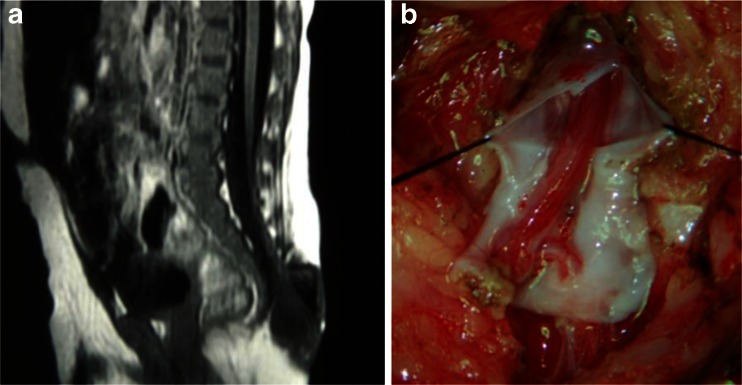
**a** A sacral meningocele: the conus medullaris is located low at the level of L5, and the fibrous band from the surface of the spinal cord herniates to the meningocele sac. **b** Intraoperative findings show that the fibrous band extends from the spinal canal to the meningocele sac

One to three kinds of lesions causing tethered cord syndrome were observed in all 43 children with low-lying conus medullaris. Twenty-one of these children had a fatty filum terminale, 13 had adhesion of the spinal cord and/or cauda equina to the base of the dural sac (Fig. 4), 5 had diastematomyelia, 3 had spinal cord lipoma, and 2 children had an arachnoid cyst. Twenty-seven children had fibrous bands extending out from the surface of the spinal cord, herniating, and growing into the inner wall of the meningocele sac (Fig. 5), or adhering to the base of the meningocele.

Lesions causing tethered cord syndrome were found during surgery in 19 of 26 children with a normally located conus medullaris; these lesions were not shown on preoperative MRI. Among these 19 children, 8 had a fibrofatty filum terminale, 8 had adhesion of the spinal cord and/or cauda equina to the base of the dural sac, and 8 had bands with one end growing on the surface of the spinal cord and the other end on the base of the meningocele. Moreover, 2 children with a normally located conus medullaris only had a meningocele with no tethered spinal cord being observed on preoperative or intraoperative MRI.

To summarize, 67 of the 69 children in this study had lesions that caused tethered cord syndrome. The most common lesions were tight filum terminale, fibrous band tethering, and adhesion of spinal cord and/or cauda equina to the dura mater. Among these 67 children, 34 had two or three other lesions besides meningocele, such as fatty filum terminale associated with spinal cord band adhesion, a spinal arachnoid cyst, or diastematomyelia. The meningocele was removed, and other lesions were treated by various surgical procedures including sectioning of the filum terminale, dissection and resection of the band adhesion, and spinal cord or cauda equina adhesiolysis. Postoperative pathological examination showed that the band was composed of fibrous tissue.

### Follow-up results

After surgery, 56 of the children who did not have preoperative symptoms were asymptomatic. Among the 10 children with sphincter dysfunction, 8 with mild symptoms recovered completely after surgery, 1 had improvement, and 1, who had serious incontinence, was lost to follow-up. Among the 3 children with foot deformities, although no improvement was observed in 2, there was no progressive aggravation and 1 child was lost to follow-up.

## Discussion

Spinal dysraphism is a birth defect caused by failed neural tube closure during development. In clinical practice, it includes many kinds of deformities, such as myeloschisis, myelomeningocele, and lipomyelomeningocele, that may cause serious neurological damage including incontinence, lower extremity paralysis, and foot deformities. Congenital meningocele, which is different from the malformations mentioned above, forms a hernial cyst that is filled with cerebrospinal fluid but does not contain neural tissue. Therefore, it has been considered to have the best prognosis, and simple meningocele repair is sufficient for preventing cyst rupture, cerebrospinal fluid leakage, and infection. In addition, it is believed that patients can live without any symptoms after surgery. In 1985, Chaseling et al. [4] performed spinal angiography on 17 children after simple meningocele repair and found related spinal cord lesions in 10 of these children; the most common lesion was a tight filum terminale. Additionally, 7 of these 10 children had symptoms caused by progressive neural damage. Recently, more and more investigators [3, 5, 6] have reported that the meningocele is often associated with tethered cord syndrome and that the incidence is as high as 90 %. Therefore, simple meningocele repair is not sufficient for the treatment of meningocele; the spinal canal should be explored, and the tethered spinal cord should be released. Otherwise, complete recovery cannot be expected even after a second surgery once tethered cord syndrome results in neurological damage, especially bladder and bowel dysfunction.

Similar to the findings of the investigators mentioned above, our findings showed that 67 of 69 children with meningocele (97 %) had other lesions which might cause tethered cord syndrome, and 34 children (49 %) had two or more lesions. The most common lesions were tight filum terminale, fibrous band tethering, and adhesion of spinal cord and/or cauda equina to the dura mater.

In the present study, MRI examination showed that 43 of 69 children had a low-lying conus medullaris, which suggested the presence of tethered cord syndrome. In addition, 5 of 26 children with a normally located conus medullaris also had spinal cord lesions. Intraoperative findings showed that low-lying conus medullaris was caused by lesions such as fatty degeneration of the filum terminale, fibrous band tethering, and spinal cord adhesion. Therefore, preoperative MRI is an important examination for children with meningocele since it can not only display the state of a meningocele but also identify potential tethered cord lesions to allow an appropriate surgical procedure to be chosen.

However, although MRI shows the lesion by multi-slice imaging and basically reflects the characteristics of the lesion, there are still certain shortcomings. In the present study, MRI displayed tethered spinal cord in 48 children. However, lesions causing tethered spinal cord were found during surgery in 19 children who did not have any lesions seen on preoperative MRI. These lesions were adhesion of spinal cord fibers, fibrous band tethering, and fibrofatty degeneration of the filum terminale. It is speculated that MRI cannot display the proliferation and adhesion of thin fibrous tissue. Therefore, intraoperative exploration of the spinal canal is extremely important. Even in patients with a normally located conus medullaris, intraoperative exploration of the spinal canal should not be ignored. It has been reported that some patients with tethered cord syndrome have a normally located conus medullaris, and the thickened filum terminale pulls the spinal cord which results in spinal cord tethering [7]. In the present study, lesions causing tethered cord syndrome were found during surgery in 19 of 26 children with a normally located conus medullaris. These lesions included fibrofatty degeneration of the filum terminale, fibrous band tethering, and spinal cord adhesion.

Erşahin et al. [3] believe that the incidence of meningocele associated with tethered cord syndrome is very high and whole-spine MRI should be performed for children with meningocele to demonstrate the tethered spinal cord. However, doctors often carry out MRI only for the affected area. If a whole-spine MRI scan has to be performed later on, the repeated examination may make children uncomfortable and make the clinical diagnosis complicated.

In the present study, all children with lumbar or sacral meningocele had associated tethered spinal cord in the lumbar or sacral region, whereas all children with thoracic meningocele had associated tethered spinal cord in the thoracic or thoracolumbar region. Because the spine of infants is short, a thoracic MRI scan in infants often can include the lumbar spine. Therefore, an MRI scan of the affected area can show the associated tethered spinal cord for children with meningocele located in the thoracic region or below. All 5 children with cervical meningocele had spinal cord band tethering or spinal cord adhesion. In addition, 1 child had associated Chari malformation. All of these lesions were located in the cervical region. Because we did not perform whole-spine MRI for our patients, we are not sure whether cervical meningocele was associated with lesions in the lumbar or sacral region. Therefore, we believe that the MRI scan of the lesion and the inferior area is sufficient for patients with thoracic or lumbosacral meningocele. For patients with cervical meningocele, we are not sure whether a cervical spine MRI is adequate or whole-spine MRI is required.

There have been different opinions about whether surgical treatment is necessary for children with asymptomatic tethered spinal cord. Some authors have suggested that surgical release of the tethered spinal cord may increase surgical trauma and risk neurological damage in affected children, and thus have advocated that only close observation should be performed for these patients [8]. Similarly, surgeons are unsure whether or not spinal canal exploration should be performed in children with asymptomatic meningocele during meningocele repair. We recommend spinal canal exploration for the treatment of meningocele and surgical release of the tethered cord for children with tethered cord syndrome regardless of the presence of symptoms because many children who do not undergo untethering of the spinal cord may develop neurological damage gradually [3, 9, 10]. In the present study, 4 children were older than 6 years of age and all of them had neurological damage caused by tethered cord syndrome. Once the neurological damage occurs, a secondary untethering of the spinal cord cannot normalize nerve function, but can only improve symptoms and prevent further aggravation of disease [2, 11, 12]. Therefore, untethering of the spinal cord should be performed during meningocele repair while the nerve is carefully protected. This is better for affected children than performing a secondary surgery to release the spinal cord after the onset of symptoms.

**Fig. 6:**
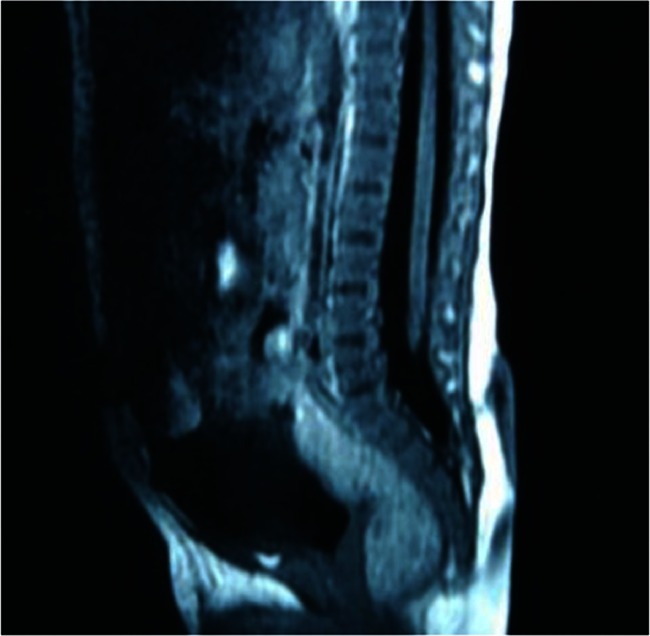
A sacral meningocele: the meningocele sac is located inferior to the conus medullaris. After the base of the meningocele sac is cut open, the filum terminale can be seen

**Fig. 7:**
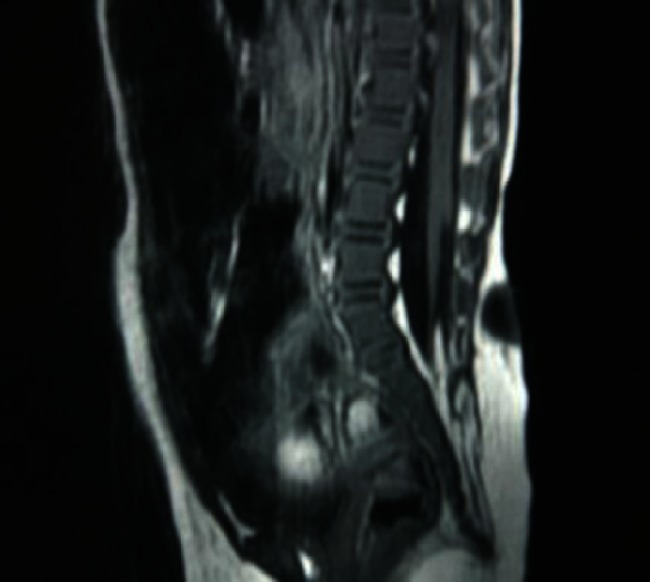
A lumbosacral meningocele: the conus medullaris is located slightly lower than the meningocele sac. A degenerated filum terminale is evident after half of the inferior spinous process has been removed

Exploration of the spinal canal and untethering of the spinal cord can be performed during meningocele repair without significant increase of surgical trauma and risk due to the following reasons: (1) the most common spinal cord lesions such as fibrous band tethering and spinal cord adhesion are usually located near the base of the meningocele sac. Therefore, the lesion can be found immediately after cutting open the base of the meningocele sac and the tethered spinal cord can then be released. (2) The meningocele is often located in the lower back. In the present study, 56 of 69 patients had the lesion in the lumbar and sacral regions. The meningocele sac was located inferior to the conus medullaris in 46 of these 56 patients. Therefore, in patients with the commonly seen tight filum terminale, the degenerated filum terminale can be found and removed immediately after cutting open the base of the meningocele sac (Fig. 6). Only in 10 children was the conus medullaris located slightly below the meningocele sac and the degenerated filum terminale could be observed after removing half or one spinous process inferiorly (Fig. 7). (3) In a few patients with diastematomyelia or arachnoid cyst, the peripheral spinous processes should be removed appropriately to expand the spinal canal for complete resection of the lesion. (4) In recent years, with the development of microsurgical techniques, in-depth understanding of the disease, and continuous improvement in surgical techniques, the safety of surgery has increased significantly, and the probability of spinal cord injury is reported to be less than 1 % when cutting off the filum terminale [13, 14]. In the present study, no spinal cord injury occurred when the spinal cord was untethered and no newly developed tethered cord syndrome was observed after surgery in any of the 69 children.

In conclusion, we believe that a meningocele is often associated with tethered cord syndrome and surgery should be performed as soon as possible for patients with or without symptoms. Preoperative MRI examination is particularly important because it can not only show the status of the meningocele sac but also demonstrate lesions causing tethered cord syndrome and provide information for choosing an appropriate surgical procedure. MRI cannot display all lesions causing tethered cord syndrome, and surgical exploration is required during meningocele repair to find lesions such as spinal cord or cauda equina adhesion, fibrous band tethering, and tight filum terminale, and the related tethered spinal cord should be released at the time of exploration.
